# Novel [1,3,4]Thiadiazole[3,2-*a*]pyrimidin-5-ones as Promising Biofilm Dispersal Agents against Relevant Gram-Positive and Gram-Negative Pathogens

**DOI:** 10.3390/md22030133

**Published:** 2024-03-15

**Authors:** Daniela Carbone, Camilla Pecoraro, Fabio Scianò, Valentina Catania, Domenico Schillaci, Barbara Manachini, Stella Cascioferro, Patrizia Diana, Barbara Parrino

**Affiliations:** 1 Department of Biological, Chemical, and Pharmaceutical Sciences and Technologies (STEBICEF), University of Palermo, Via Archirafi 32, 90123 Palermo, Italy; daniela.carbone@unipa.it (D.C.); camilla.pecoraro@unipa.it (C.P.); fabio.sciano@unipa.it (F.S.); domenico.schillaci@unipa.it (D.S.); patrizia.diana@unipa.it (P.D.); barbara.parrino@unipa.it (B.P.); 2 Department of Earth and Marine Sciences (DiSTeM), University of Palermo, Viale delle Scienze Ed. 16, 90128 Palermo, Italy; valentina.catania@unipa.it; 3 Department of Agricultural, Food and Forest Sciences (SAAF), University of Palermo, Viale delle Scienze 13, 90128 Palermo, Italy; barbara.manachini@unipa.it

**Keywords:** nortopsentin analogs, anti-biofilm agents, thiadiazopyrimidinone derivatives, dispersal activity, *Galleria mellonella*

## Abstract

Biofilm-associated infections pose significant challenges in healthcare settings due to their resistance to conventional antimicrobial therapies. In the last decade, the marine environment has been a precious source of bioactive molecules, including numerous derivatives with antibiofilm activity. In this study, we reported the synthesis and the biological evaluation of a new series of twenty-two thiadiazopyrimidinone derivatives obtained by using a hybridization approach combining relevant chemical features of two important classes of marine compounds: nortopsentin analogues and Essramycin derivatives. The synthesized compounds were in vitro tested for their ability to inhibit biofilm formation and to disrupt mature biofilm in various bacterial strains. Among the tested compounds, derivative **8j** exhibited remarkable dispersal activity against preformed biofilms of relevant Gram-positive and Gram-negative pathogens, as well as towards the fungus *Candida albicans*, showing BIC_50_ values ranging from 17 to 40 µg/mL. Furthermore, compound **8j** was in vivo assayed for its toxicity and the anti-infective effect in a *Galleria mellonella* model. The results revealed a promising combination of anti-infective properties and a favorable toxicity profile for the treatment of severe chronic biofilm-mediated infections.

## 1. Introduction

The emergence of bacterial strains resistant to commonly used antibiotic therapies has posed a significant global health threat, leading to profound economic and social implications [1,2]. Unfortunately, the situation has further exacerbated in recent years due to the COVID-19 pandemic, resulting in a sharp rise in cases of multidrug-resistant (MDR) infections [3]. This surge is believed to be largely attributed to the excessive and inappropriate use of antibiotics during the initial phase of the pandemic. Chronic infections caused by antibiotic-resistant strains have emerged as a leading cause of mortality across all age groups [4]. The rise in these infections poses a significant threat to public health, necessitating urgent attention and innovative approaches to combat the growing crisis.

The ability of bacteria to grow in aggregate structures, called biofilms, has played a significant role in the widespread occurrence of antibiotic resistance [5]. Bacterial biofilms are considered crucial virulence factors that greatly contribute to pathogen survival in hostile environments [6]. Apart from providing physical protection through the matrix, which shields the pathogens from antibiotics, bacterial cells within biofilms possess the same resistance mechanisms found in their planktonic counterparts, such as enzymatic resistance, alterations in cell permeability, efflux pumps, and modifications of the antibiotic target [7]. Furthermore, the deepest layers of biofilms harbor metabolically inactive cells, known as persistent or dormant cells, which intrinsically display resistance to common antibiotic therapies [8]. Cumulatively, these mechanisms render bacterial cells within biofilms approximately 1000 times more resistant than their planktonic counterparts, making biofilm-associated infections extremely challenging to treat [9]. It is estimated that approximately 80% of chronic infections are directly linked to biofilm formation [10].

**Figure 1:**
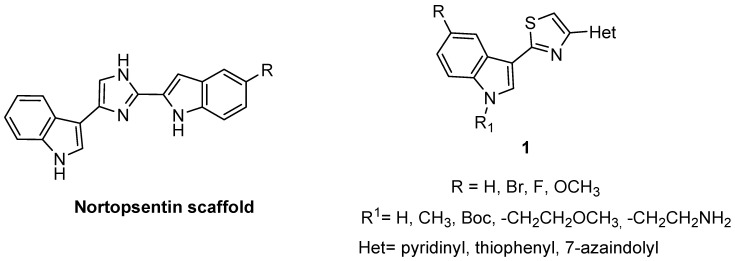
Chemical structure of thiazole nortopsentin analogs of type **1** with anti-biofilm activity.

In recent years, significant efforts have been dedicated to the search for small molecules that can effectively target the biofilm lifestyle by either preventing its formation or disrupting the architecture of mature biofilms [11,12,13]. The marine environment is a precious source of derivatives endowed with antibiofilm activity, and many marine derivatives have been employed as lead compounds for the development of new molecules that are able to interfere with this complicated bacterial form of life [14]. In recent years, structural manipulation of the marine alkaloid nortopsentin, extracted from the sponge *Spongsorites ruetzleri*, led us to obtain potent antivirulence derivatives with an antibiofilm mechanism of action, which are able to interfere with the first step of the biofilm formation [15]. Among the different synthesized analogues, nortopsentin derivatives of type **1** (Figure 1), in which the imidazole nucleus of the marine alkaloid was replaced by the thiazole ring, whose position 5 was substituted with different heterocycles, including 7-azaindole, thiophene, and pyridine, showed a potent activity in inhibiting biofilm formation [15].

In particular, the thiazole nortopsentin analogues that were synthesized exhibited a marked selectivity against Gram-positive pathogens, eliciting, in some cases, IC_50_ values against *Staphylococcus aureus* ATCC 25923 lower than 1 µM.

**Figure 2:**
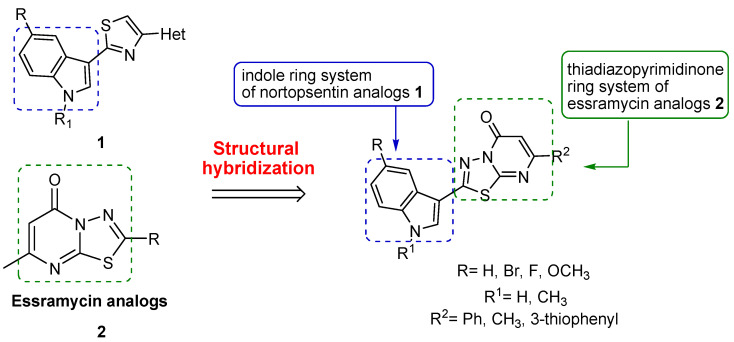
Structural manipulation of thiazole nortopsentin library.

In order to deepen the SAR of this class of nortopsentin derivatives and in an attempt to obtain more potent anti-biofilm agents, we decided to further investigate the substitution of the central scaffold. Considering the interesting antimicrobial activities described for several compounds containing multisubstituted or condensed pyrimidine structures [16], such as the Essramycin thiadiazoanalogues of type **2** (Figure 2) [17,18], we decided to synthesize new indole compounds bearing the [1,3,4]thiadiazolo[3,2-*a*]pyrimidin-5-one system as central moiety.

## 2. Results and Discussion

### 2.1. Chemistry

**Scheme 1:**
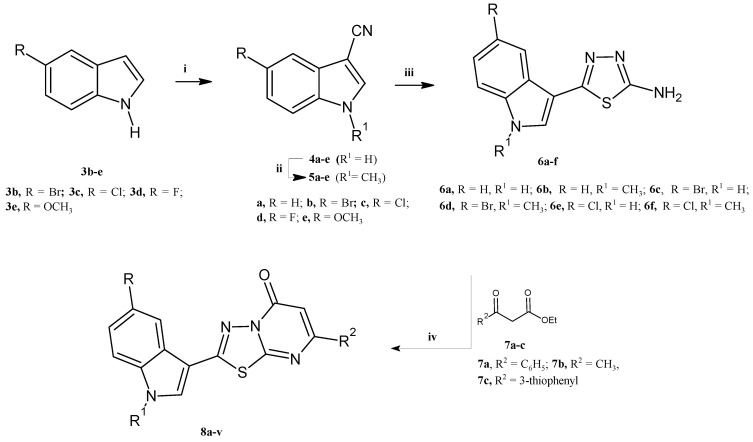
Synthesis of the new [1,3,4]thiadiazole[3,2-a]pyrimidin-5-ones **8a**–**v**. Reagents and conditions: (i) CH_3_CN, CSI, 0 °C, 2 h, then DMF, 0 °C, 1.5 h (98–100%); (ii) DMF, (CH_3_O)_2_CO, K_2_CO_3_, 130 °C, 3.5 h (98–100%); (iii) trifluoroacetic acid, thiosemicarbazide, 60 °C, 3.5 h (98–100%); (iv) PPA, 130 °C, 2 h (50–75%).

A series of twenty-two[1,3,4]thiadiazole[3,2-a]pyrimidin-5-one derivatives **8a**–**v** (Table 1) were successfully synthesized by the reaction of the aminothiadiazole intermediate **6** with the appropriate β-ketoester. The reaction was carried out in polyphosphoric acid (PPA) under optimized conditions, as depicted in Scheme 1.

The carbonitriles **4** and their methylated derivatives **5**, required for the synthesis of the key intermediates **6**, were obtained from the reaction of the appropriate 1H-indoles **3** with chlorosulfonyl isocyanate (CSI) in anhydrous acetonitrile under stirring at 0 °C and subsequent treatment with dimethyl carbonate in anhydrous DMF under reflux at 130 °C, as previously described [19]. The reaction of indoles **4** or **5** with thiosemicarbazide in trifluoroacetic acid (TFA) at 60 °C for about 3 h led to the desired intermediates **6**.

The β-ketoesters **7a**,**b,** employed in the final reaction, are commercially available, whereas derivative **7c** was prepared by 3-acetylthiophene treated with NaH and diethyl carbonate in DMF.

### 2.2. Biology

#### 2.2.1. Antibacterial Activity

The newly synthesized thiadiazopyrimidinones **8a**–**v** were tested for their in vitro antibacterial activity against the planktonic form of the Gram-positive pathogens *S. aureus* ATCC 25923 and Enterococcus faecalis ATCC 29212, as well as the Gram-negative *P. aeruginosa* ATCC 15442 and *E. coli* ATCC 25922. Most of the tested compounds displayed minimum inhibitory concentration (MIC) values exceeding 100 µg/mL, indicating limited antibacterial activity. However, a subset of derivatives, specifically **8e**, **8f**, **8j**, **8k**, and **8l**, exhibited lower MIC values against tested Gram-positive strains, which are summarized in Table 2.

While higher MIC values may appear discouraging in terms of antibacterial activity, they can actually be advantageous when aiming to develop compounds with an anti-virulence profile. Such compounds have the ability to inhibit critical bacterial virulence factors without affecting the growth or viability of the pathogens [19]. Therefore, the derivatives exhibiting higher MIC values in this study may hold potential as anti-virulence agents, which could be further explored to assess their efficacy in targeting specific virulence mechanisms of the tested pathogens. The anti-virulence strategy is considered an advantageous approach, since it imposes limited selective pressure in promoting the development of the antibiotic-resistance mechanisms. Among the different virulence factors used by pathogens to cause diseases, biofilm formation is currently considered one of the most relevant, strongly contributing to the microbial resistance to common antibiotic therapies.

#### 2.2.2. Inhibition of Biofilm Formation

Compounds **8a**–**v** were then tested in vitro at sub-MIC concentrations with the aim of evaluating their ability to inhibit biofilm formation against the same tested strains. Derivatives **8a**,**b**,**d**,**g**,**h**,**i**,**m**–**v** elicited percentage values of inhibition ranging from 20 to 33%. For the compounds that showed a percentage of inhibition greater than 50% at the screening concentration, further tests were carried out at lower concentrations to establish the BIC_50_ values, which are the concentrations at which the percentage of inhibition of biofilm formation is equal to 50% compared to the untreated growth control. Among the new compounds, thiadiazopyrimidinones **8c**,**e**,**f**,**j**,**k**,**l** proved to be active in inhibiting the biofilm formation in at least one of the tested strains and the BIC_50_ values are reported in Table 3.

In comparison to the previously reported imidazothiadiazole compounds **1**, the new derivatives exhibited significantly reduced activity in inhibiting biofilm formation. Only compounds **8f**, **8j**, and **8k** demonstrated notable anti-biofilm activity. Specifically, compounds **8f** and **8k** displayed BIC_50_ values of 7.6 µg/mL (21.3 µM) and 6.4 µg/mL (22.6 µM) against *S. aureus* ATCC 25923 and E. faecalis ATCC 29212, respectively. Compound **8j**, on the other hand, hindered the biofilm formation process in all tested bacterial strains, except E. faecalis, with BIC_50_ values ranging from 6.1 to 14.4 µg/mL (16.2–38.4 µM).

#### 2.2.3. Dispersal Activity against Pre-Formed Biofilm

Compounds **8a**–**l**, belonging to the most potent subset, substituted in position 7 of the thiadiazopyrimidinone scaffold with a phenyl or methyl group, were selected for further assays in order to evaluate their dispersal activity against 24 h preformed biofilms of *S. aureus* ATCC 25923, *P. aeruginosa* ATCC 15442, and *E. coli* ATCC, which have been shown to be more sensitive to the compounds. The percentages of inhibition observed at the concentration of 200 µg/mL are reported in Table 4.

Interestingly, all compounds were proved to be able to disrupt the biofilm architecture of Gram-positive and Gram-negative pathogens, exhibiting inhibition percentages ranging from 51 to 77% at 200 µg/mL. Additional experiments were carried out at lower concentrations in order to define the BIC_50_ values as dispersal agents.

In particular, derivatives **8c** and **8j** showed BIC_50_ values lower than 150 µg/mL against all three tested strains, while derivative **8c** displayed IC_50_ values of 141 µg/mL (333 µM) against the Gram-positive *S. aureus* and 126 and 120 µg/mL (297 and 283 µM) against the Gram-negative pathogens *P. aeruginosa* ATCC 15442 and *E. coli* ATCC 25922, respectively.

Interestingly, compound **8j** maintained high potency at lower concentrations, showing BIC_50_ values of 27, 21 and 17 µg/mL (71, 55 and 45 µM) against *S. aureus* ATCC 25923, *P. aeruginosa* ATCC 15442 and *E. coli* ATCC 25922, respectively.

Considering the promising activity of derivative **8j**, its dispersal properties were evaluated against other biofilm-forming microorganisms, including the dermatological Gram-positive pathogens Propionibacterium acnes and Staphylococcus homnis, and the fungus Candida albicans. The thiadiazopyrimidinone **8j** also proved to be very potent against these microorganisms, exhibiting BIC_50_ values of 39, 36, and 40 µg/mL (103, 95, and 106 µM), respectively. These results sound very encouraging, not only in terms of the potency of their dispersal activity but also in terms of their spectrum of action, since compound **8j** showed very high potency in disrupting biofilm architecture in all tested strains.

The biological data that were obtained highlighted that, among the examined substitutions, the one that had the most influence on the antibiofilm activity is the one in position 7 of the [1,3,4]thiadiazolo[3,2-a]pyrimidinone scaffold. The presence of a phenyl ring (**8c**,**f**,**j**) or a methyl group (**8k**,**l**) in this position proved to be advantageous for the activity against the bacterial biofilm form of life. Conversely, the substitution in position 7 with a thiophene ring appeared detrimental to the anti-biofilm properties of this class of compounds. Additionally, the dispersal activity also seems to benefit from the presence of a halogen, such as a bromine or fluorine atom (**8c** and **8j**), in position 5 of the indole nucleus.

#### 2.2.4. In Vivo Anti-Infective Evaluation of **8j**

For the in vivo studies, we decided to employ Galleria mellonella larvae. The use of *G*. *mellonella* larvae as an in vivo model in pre-clinical research has gained popularity due to several factors. Firstly, the larvae share numerous similarities with “standard” animal models, including the presence of a humoral and cellular immune system. Additionally, the unique characteristics of *G*. *mellonella*, such as its larval size, ease of rearing, and ability to survive even at 37 °C, contribute to the success of this species as an alternative animal model to mammals. These advantages make it a practical and cost-effective option for initial toxicity assessments and drug efficacy studies [20,21].

**Figure 3:**
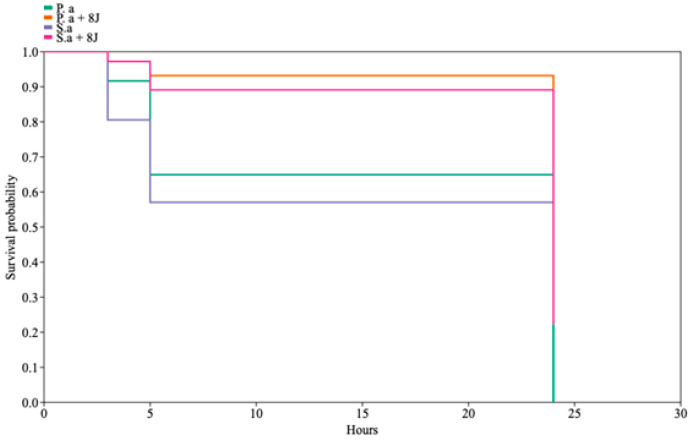
Survivorship of Galleria mellonella larvae infected with *P. aeruginosa* (P.a, green line) and *S. aureus* (S.a, purple line) and larvae infected with P.a. and S.a. and injected with the compound **8j** (1 mg/kg) (orange and pink lines, respectively). Values were determined by the Kaplan–Meier method and compared using the log-rank test (x^2^ = 9.34, *p* = 0.002).

The protective effect of compound **8j** was evaluated in vivo using *G*. *mellonella* larvae inoculated with *P. aeruginosa* or *S. aureus*. These two bacteria were selected because of their relevance as pathogens and antibiotic resistant strains and, importantly, they are reported to be entomopathogens infecting *G*. *mellonella* and other insects [22]. The survival of infected larvae, not treated and treated with the compound **8j** (1 mg/kg), was compared in order to evaluate the protective effect of our derivative. Although the two pathogens showed the same level of pathogenicity (*p* > 0.05), compound **8j** was more effective against *P. aeruginosa* than *S. aureus* after 6 h of treatment (*p* = 0.039) and over all experiments (*p* = 0.049). A survival analysis of larvae infected by *P. aeruginosa* and *S. aureus* and treated with compound **8j** revealed significant differences between treated and untreated groups (x^2^ = 20.39, DF = 4, *p* < 0.001) (Figure 3), indicating an interesting protective effect of compound **8j**. After 20 h, survival was about 90% in larvae treated with **8j**, declining to about 60% and 50% for untreated larvae infected by *P. aeruginosa* and *S. aureus,* respectively.

#### 2.2.5. Toxicity Evaluation of **8j** in In Vivo Model

**Figure 4:**
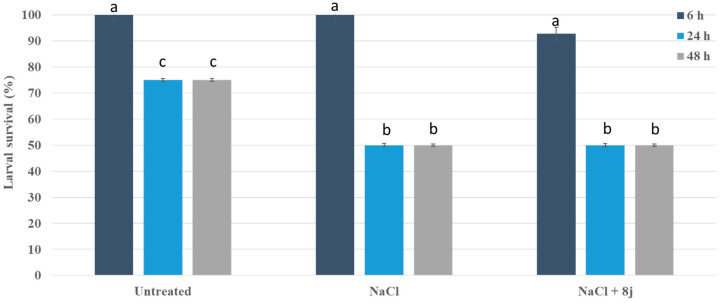
Effect of treatment with compound **8j** on survival of *G*. *mellonella* larvae after 6, 24, and 48 h compared with not treated and treated with solvent (NaCl 0.9%) larvae. Significant differences (*p* < 0.05) among the mortality are reported and indicated with different letters.

The toxicity of the most active derivative, compound **8j**, was assessed in vivo in *G*. *mellonella* larvae. A single dose of 1 mg/kg, dissolved in NaCl 0.9% (*v*/*v*), was administered to groups of 12 larvae. The effects were observed at 6, 24, and 48 h post-treatment. The toxicity assessment was based on the percentage of larval survival (Figure 4). By monitoring the survival percentage, the potential adverse effects of compound **8j** were evaluated in this model. The results provide insights into the compound’s safety profile and its potential suitability for further development as a therapeutic agent.

The comparison of the survival percentages between the group of larvae treated with compound **8j** and the control group (which only received injections of the solvent) indicates the absence of toxicity at the examined dose and regimen throughout the experiment. A slight increase mortality compared to the larvae treated with only NaCl was recorded only 6 h after the treatment but was not statistically significant (*p* > 0.05). This finding suggests that compound **8j** is well-tolerated and does not exert harmful effects on *G*. *mellonella* larvae under the conditions of this study.

## 3. Material and Methods

### 3.1. Chemistry

The anhydrous solvents used for organic synthesis and the reagents were purchased from Sigma-Aldrich Co. (St. Louis, MO, USA), Alfa Aesar (Haverhill, MA, USA), VWR International (Radnor, PA, USA), and Acros Organics (Waltham, MA, USA). All air- or moisture-sensitive reactions were carried out using oven-dried glassware under an inert dry nitrogen atmosphere. Analytical thin-layer chromatography (TLC) was performed on silica gel 60 F254 plates (0.25 mm thickness) and the developed plates were examined under ultraviolet (UV) light. All melting points were taken on a Buchi–Tottoly (Büchi, Cornaredo, Italy) capillary apparatus and were uncorrected. IR spectra were determined in bromoform with a Shimadzu FT/IR 8400S spectrophotometer, and peaks were reported in wavenumber (cm^−1^). 1H and 13C NMR spectra were measured at 200 and 50 MHz, respectively, on DMSO-d6 solution, using a Bruker Avance II series 200 MHz spectrometer. Chemical shifts were described in parts per million (δ), coupling constants (J) were expressed in Hertz (Hz), and splitting patterns were reported as singlet (s), doublet (d), triplet (t), quartet (q), multiplet (m), doublet of doublets (dd), and triplet of doublets (td). A chromatography column was performed with MERK silica gel 230–400 mesh ASTM or FLASH40i Biotage chromatography or with Buchi Sepacore chromatography module (prepacked cartridge reference).

#### 3.1.1. Synthesis of 1H-indole-3-carbonitriles (**4b**–**e**)

Compounds **4b**–**e** were obtained using a solution of the suitable indole **3** (5.10 mmol) in anhydrous acetonitrile (4.5 mL) treated dropwise with chlorosulfonyl isocyanate (CSI) (0.44 mL, 5.10 mmol). After keeping the mixture at 0 °C under stirring for 2 h, anhydrous dimethylformamide (DMF) (2.8 mL, 36.39 mmol) was slowly added. The mixture was stirred at 0 °C for 1.5 h, and the resulting solution was poured into crushed ice, giving a solid, which was then filtered and dried (yields 98–100%). Analytical and spectroscopic data for compounds **4b**–**e** are in agreement with those previously reported [23].

#### 3.1.2. Synthesis of 1-methylindole-3-carbonitriles (**5a**–**e**)

A total of 3.61 mmol of K_2_CO_3_ and 21.4 mmol of dimethyl carbonate (1.8 mL) were added to a solution of the appropriate 3-cyanoindole 4 (7.03 mmol) in anhydrous DMF (10 mL), and the reaction mixture was heated at 130 °C for 3.5 h. After cooling (0–5 °C), water and ice (25 mL) were slowly added under stirring, leading to the formation of an oily suspension, which was extracted with diethyl ether (3 × 10 mL). The organic phase was washed with water and brine and dried over Na_2_SO_4_, and the solvent evaporated at reduced pressure to obtain the 3-cyano-1-methylindoles **5** in excellent yields. Analytical and spectroscopic data are in accordance with those reported in the literature [11].

#### 3.1.3. Synthesis of 5-(1H-indol-3-yl)-1,3,4-thiadiazol-2-amines (**6a**–**f**)

A mixture of the suitable indole-3-carbonitrile **4a**–**e** or **5a**–**e** (5 mmol), thiosemicarbazide (5 mmol), and trifluoroacetic acid (5 mL) was heated under stirring at 60 °C for 3.5 h. The reaction mixture was then poured into ice and neutralized with a saturated solution of NaHCO_3_. The resulting solid was filtered, and washed with water, cyclohexane, and diethyl ether to yield 5-(1H-indol-3-yl)-1,3,4-thiadiazol-2-amines **6a**–**f** in excellent yields. The analytical and spectroscopic data for the derivatives **6a**–**f** are consistent with those reported in the literature [11].

#### 3.1.4. Synthesis of Ethyl 3-oxo-3-(thiophen-3-yl)propanoate **7c**

A solution of 2.3 mmol of 3-acetylthiophene in 2.3 mL of THF was added dropwise to a mixture of 110.4 mg of NaH in 3.68 mL of anhydrous THF under a nitrogen atmosphere. The addition was carried out over a period of 20 min while stirring at room temperature. A slight increase in temperature (4–5 °C) was observed during the addition.

The reaction mixture was warmed to 35 °C and stirred for 30 min. A THF solution of diethyl carbonate (4.6 mmol) was then added slowly over a period of 1 h. After an additional hour, the reaction mixture was cooled down to −10 °C and quenched by slowly adding water (5–10 mL). After the addition of glacial acetic acid (0.27 mL), the mixture was stirred for 20 min and then warmed to room temperature. The organic layer was separated, and the aqueous layer was extracted three times with ethyl acetate (20 mL each time). The combined organic layers were washed with brine, dried with anhydrous Na_2_SO_4_, and concentrated under reduced pressure.

The resulting compound **7c** was purified using column chromatography with a silica gel column. A mixture of hexanes and ethyl acetate was used as the eluent. Cyclohexane (100%) was initially employed to elute the excess diethyl carbonate, and then the amount of ethyl acetate in the eluent was progressively increased from 20% to 50% in order to elute the desired compound.

The analytical and spectroscopic data for derivative **7** were found to be consistent with those reported in the literature.

#### 3.1.5. General Procedure for the Synthesis of -[1,3,4]thiadiazolo[3,2-a]pyrimidin-5-ones (**8a**–**v**)

Equimolar amounts of 5-(1H-indol-3-yl)-1,3,4-thiadiazol-2-amine **6a**–**f** (0.92 mmol) and the suitable β-ketoester **7** (0.92 mmol) were stirred in PPA (1 g) at 130 °C for 2 h. After cooling to room temperature, the reaction was quenched by adding ice-cold water, and the resulting solid was filtered. The product was then treated with saturated NaHCO_3_ aqueous solution, and the crude was purified by column chromatography.

2. -(1H-Indol-3-yl)-7-phenyl-5H-[1,3,4]thiadiazolo[3,2-a]pyrimidin-5-one (**8a**)

Yield: 75%; white solid; mp: 321 °C; IR (cm^−1^): 3142 (NH);1653 (CO) ^1^H NMR (200 MHz, DMSO) δ 7.03 (1H, d, J = 2.0 Hz, Ar); 7.32 (2H, dd, J = 2.6, 3.1 Hz, Ar), 7.32–7.57 (4H, m, Ar), 8.13 (2H, dd, J = 1.3, 2.7 Hz, Ar); 8.24 (1H, dd, J = 2.4, 3.3 Hz, Ar); 8.41 (1H, d, J = 2.3 Hz, Ar), 12.27 (1H, s, NH). ^13^C NMR (50 MHz, DMSO) δ: 103.9 (d), 105.9 (s), 113.1 (d), 121.2 (d), 122.3 (d), 123.9 (d), 124.2 (s), 127.5 (2xd), 129.3 (2xd), 131.2 (d), 132.0 (d), 136.0 (s), 137.3 (s), 154.1 (s), 157.0 (s), 159.0 (s), 161.6 (s). Anal. Calculated for C_19_H_12_N_4_OS (MW: 344.39): C, 66.26; H, 3.51%; N, 16.27%. Found: C, 66.53; H, 3.74%; N, 16.38%.

2. -(5-Methoxy-1H-indol-3-yl)-7-phenyl-5H-[1,3,4]thiadiazolo[3,2-a]pyrimidin-5-one (**8b**)

Yield: 61%; brown solid; mp: 328 °C; IR (cm^−1^): 3207 (NH); 1662 (CO) ^1^H NMR (200 MHz, DMSO) δ: 3.85 (3H, s, CH_3_), 6.69 (1H, d, J = 8.9 Hz, Ar), 7.02 (1H, s, Ar), 7.47 (1H, d, J = 9.1 Hz, Ar), 7.49–7.53 (3H, m, Ar), 7.73 (1H, s, Ar), 8.14 (2H, dd, J = 1.7, 3.3 Hz, Ar), 8.35 (1H, s, Ar), 12.17 (1H, s, NH).^13^C NMR (50 MHz, DMSO) δ: 55.9 (q), 100.6 (s), 103.5 (d), 103.9 (d), 105.6 (s), 113.3 (d), 113.8 (d), 124.9 (s), 127.6 (d), 129.2 (d), 129.4 (d), 131.1 (d), 132.2 (s), 136.0 (s), 142.8 (d), 154.0 (s), 155.8 (s), 156.9 (s), 157.1 (d), 158.9 (s). Anal. Calculated for C_20_H_14_N_4_O_2_S (MW: 374.41): C, 64.16; H, 3.77; N, 14.96%. Found: C, 64.36; H, 3.91; N, 14.85%.

2. -(5-Bromo-1H-indol-3-yl)-7-phenyl-5H-[1,3,4]thiadiazolo[3,2-a]pyrimidin-5-one (**8c**)

Yield: 68%; yellow solid; mp: 348 °C; IR (cm^−1^): 3170 (NH); 1660 (CO) ^1^H NMR (200 MHz, DMSO) δ: 7.04 (1H, s, Ar), 7.44 (1H, d, J = 8.3 Hz, Ar), 7.52 (4H, m, Ar), 8.13 (2H, d, J = 2.8 Hz, Ar), 8.36 (1H, s, Ar), 8.47 (1H, s, Ar), 12.46 (1H, s, NH). ^13^C NMR (50 MHz, DMSO) δ: 103.9 (d), 105.5 (s), 114.9 (s), 115.2 (d), 123.3 (d), 125.8 (s), 126.6 (d), 127.5 (2xd), 129.3 (2xd), 131.2 (d), 133.3 (d), 135.9 (s), 136.0 (s), 153.7 (s), 157.0 (s), 159.1 (s), 161.5 (s). Anal. Calculated for C1_9_H_11_BrN_4_OS (MW: 423.28): C, 53.91; H, 2.62; N, 13.24%. Found: C, 55.71; H, 2.91; N, 13.50%.

2. -(5-Chloro-1H-indol-3-yl)-7-phenyl-5H-[1,3,4]thiadiazolo[3,2-a]pyrimidin-5-one (**8d**)

Yield: 50%; beige solid; mp: 320 °C; IR (cm^−1^): 3134 (NH); 1663 (CO) ^1^H NMR (200 MHz, DMSO) δ 7.02 (1H, s, Ar), 7.32 (1H, d, J = 4.3 Hz, Ar),7.51–7.57 (4H, m, Ar), 8.11 (2H, m, Ar); 8.19 (1H, d, J = 3.8 Hz, Ar), 8.46 (1H, s, Ar), 12,42 (1H, s, NH). ^13^C NMR (50 MHz, DMSO) δ: 103.9 (d), 105.6 (s), 114.8 (d), 120.3 (d), 124.0 (d), 125.2 (s), 126.9 (s), 127.5 (2xd), 129.3 (2xd), 131.2 (s), 133.5 (d), 135.8 (s), 135.9 (s), 139.3 (d), 153.7 (s), 157.0 (s), 159.1 (s). Anal. Calculated for C_19_H_11_ClN_4_OS (MW: 378.83): C, 60.24; H, 2.93; N, 14.79%. Found: C, 60.66; H, 3.11; N, 14.90%.

2. -(5-Fluoro-1H-indol-3-yl)-7-phenyl-5H-[1,3,4]thiadiazolo[3,2-a]pyrimidin-5-one (**8e**)

Yield: 62%; pale yellow solid; mp > 350 °C; IR (cm^−1^): 3230 (NH); 1678 (CO); ^1^H NMR (200 MHz, DMSO) δ 7.04 (1H, d, J = 3.0 Hz, Ar), 7.19 (1H, d, J = 1.3, Ar), 7.41–7.64 (4H, m, Ar), 7.94 (1H, d, J = 3.2, Ar), 8.14 (2H, dd, J = 1.2, 1.5 Hz, Ar), 8.48 (1H, d, J = 1.5 Hz, Ar), 12.38 (1H, s, NH).^13^C NMR (50 MHz, DMSO) δ: 103.96 (d), 109.59 (s), 112.07 (d), 123.40 (s), 127.53 (d), 129.30 (d), 129.69 (d), 131.20 (s), 133.91 (d), 134.24 (s), 135.97 (d), 137.13 (s), 148.97 (d), 153.88 (d), 157.02(s), 159.11 (d), 159.53 (s), 161.34 (s), 170.41 (s). Anal. Calculated for C_19_H_11_FN_4_OS (MW: 362.38): C, 62.97; H, 3.06; N, 15.46%. Found: C, 63.13; H, 3.32; N, 15.59%.

2. -(1-Methyl-1H-indol-3-yl)-7-phenyl-5H-[1,3,4]thiadiazolo[3,2-a]pyrimidin-5-one (**8f**)

Yield: 56%; light brown solid; mp: 265 °C; IR (cm^−1^): 1678 (CO). ^1^H NMR (200 MHz, DMSO) δ 3.9 (3H, s, CH_3_), 6.99 (1H, s, Ar), 7.34–7.51 (5H, m, Ar), 7.6 (1H, d, J = 4.6 Hz, Ar), 8.09 (1H, s, Ar), 8.22 (2H, dd, J = 3.4, 3.3 Hz, Ar), 8.37 (1H, s, Ar).^13^C NMR (50 MHz, DMSO) δ: 33.72 (q), 103.95 (d), 104.77 (s), 111.57 (d), 121.29 (d), 122.63 (d), 123.93 (d), 124.53 (s), 127.5 (2xd), 129.26 (2xd), 131.15 (d), 135.29 (d), 135.97 (s), 137.81 (s), 153.54 (s), 156.96 (s), 158.96 (s), 161.42 (s). Anal. Calculated for C_20_H_14_N_4_OS (MW: 358.41): C, 67.02; H, 3.94; N, 15.63%. Found: C, 67.31; H, 4.21; N, 15.79%.

2. -(5-Methoxy-1-methyl-1H-indol-3-yl)-7-phenyl-5H-[1,3,4]thiadiazolo[3,2-a]pyrimidin-5-one (**8g**)

Yield: 54%; beige solid; mp: 335 °C; IR (cm^−1^): 1678 (CO). ^1^H NMR (200 MHz, DMSO) δ: 3.87 (3H, s, CH_3_), 3.90 (3H, s, CH3), 7.02 (2H, s, Ar), 7.54 (4H, m, Ar), 7.71 (1H, d, J = 2.5 Hz, Ar), 8.12–8.15 (2H, m, Ar), 8.37 (1H, s, Ar).^13^C NMR (50 MHz, DMSO) δ: 33.9 (q), 56.0 (q), 103.7 (d), 103.9 (d), 104.3 (s), 112.5 (d), 113.2 (d), 125.3 (2xs), 127.52 (2xd), 129.31 (2xd), 131.1 (d), 133.0 (d), 135.4 (2xs), 136.0 (s), 153.6 (s), 156.2 (s), 156.9 (s), 158.9 (s). Anal. Calculated for C_21_H_16_N_4_O_2_S (MW: 388.44): C, 64.93; H, 4.15; N, 14.42%. Found: C, 64.80; H, 4.35; N, 14.70%.

2. -(5-Bromo-1-methyl-1H-indol-3-yl)-7-phenyl-[1,3,4]thiadiazolo[3,2-a]pyrimidin-5-one (**8h**)

Yield: 61%; off-white solid; mp: 318 °C; IR (cm^−1^): 1663 (CO). ^1^H NMR (200 MHz, DMSO) δ: 3.9 (3H, s, CH_3_), 7.03 (1H, s, Ar), 7.53–7.65 (4H, m, Ar), 7.64 (1H, d, J = 8.6 Hz, Ar), 8.13 (2H, dd, J = 1.8, 3.5 Hz, Ar), 8.34 (1H, s, Ar), 8,47 (1H, s, Ar). ^13^C NMR (50 MHz, DMSO) δ: 34.0 (q), 104.0 (d), 104.3 (s), 113.9 (d), 115.4 (s), 116.4 (s), 123.4 (d), 123.4 (d), 126.6 (d), 127.5 (d), 127.5 (d), 129.3 (d), 130.0 (d), 134.6 (s), 135.8 (d), 136.7 (s), 142.5 (s), 151.9 (s), 155.5 (s), 171.1 (s). Anal. Calculated for C_20_H_13_BrN_4_OS (MW: 437.31): C, 54.93; H, 3.00; N, 12.81%. Found: C, 54.81; H, 3.26; N, 12.95%.

2. -(5-Chloro-1-methyl-1H-indol-3-yl)-7-phenyl-5H-[1,3,4]thiadiazolo[3,2-a]pyrimidin-5-one (**8i**)

Yield: 62%; off-white solid; mp: 312 °C; IR (cm^−1^): 1676 (CO). ^1^H NMR (200 MHz, DMSO) δ 3.91 (3H, s, CH_3_), 7.02 (1H, s, Ar), 7.39 (1H, d, J = 8.7 Hz, Ar), 7.47 (3H, m, Ar), 7.67 (1H, d, J = 6.7 Hz, Ar), 8.14 (2H, dd, J = 2.4, 3.3 Hz, Ar), 8.2 (1H, s, Ar), 8.46 (1H, s, Ar). ^13^C NMR (50 MHz, DMSO) δ 33.4 (q), 104.0 (d), 105.2 (s), 112.6 (d), 116.1 (s), 120.6 (d), 122.9 (d), 125.8 (s), 125.9 (d), 127.4 (s), 127.5 (d), 128.4 (d), 128.9 (d), 129.3 (d), 129.3 (d), 131.6 (s), 135.9 (s), 136.4 (s), 168.1 (s), 188.0 (s). Anal. Calculated for C_20_H_13_ClN_4_OS (MW 392.86): C, 61.14; H, 3.34; N, 14.26%. Found: C, 61.38; H, 3.52; N, 14.40%.

2. -(5-Fluoro-1-methyl-1H-indol-3-yl)-7-phenyl-5H-[1,3,4]thiadiazolo[3,2-a]pyrimidin-5-one (**8j**)

Yield: 65%; pale yellow solid; mp: 299 °C; IR (cm^−1^): 1681 (CO). ^1^H NMR (200 MHz, DMSO) δ 3.89 (3H, s, CH_3_), 6.98 (1H, s, Ar), 7.2–7.57 (4H, m, Ar); 7.61 (1H, d, J = 2.7 Hz, Ar), 7.87 (2H, dd, J = 2.3, 8.8, Ar); 8.43(1H, s, Ar).^13^C NMR (75MHz, DMSO) δ: 34.0 (q), 103.9 (d), 104.7 (s), 106.0 (d), 106.4 (d), 112.0 (d), 112.3 (d), 113.3 (d), 124.8 (s), 127.5 (d), 129.2 (d), 131.2 (d), 134.5 (s), 135.9 (s), 136.8 (d), 153.3 (s), 156.9 (s), 157.6 (s), 159.0 (s), 160.7 (s). Anal. Calculated for C_20_H_13_FN_4_OS (MW: 376.41): C, 63.82; H, 3.48; N, 14.88%. Found: C, 63.97; H, 3.65; N, 15.05%.

2. -(1H-Indol-3-yl)-7-methyl-5H-[1,3,4]thiadiazolo[3,2-a]pyrimidin-5-one (**8k**)

Yield: 58%; off-white solid; mp: 271 °C; IR (cm^−1^): 3360 (NH), 1674 (CO). ^1^H NMR (200 MHz, DMSO) δ 2.29 (3H, s, CH_3_), 6.29 (1H, s, Ar), 7.11–7.33 (2H, m, Ar), 7.48 (1H, dd, J = 1.7, 6.5 Hz, Ar), 8.19 (1H, dd, J = 3.4, 3.3 Hz, Ar), 8.36 (1H, s, Ar), 12.23 (1H, s, NH). ^13^C NMR (50 MHz, DMSO) δ: 23.7 (q), 105.8 (s), 107.0 (d), 107.2 (s), 113.0 (d), 121.2 (d), 122.3 (d), 123.9 (d), 124.8 (s), 131.8 (d), 137.2 (s), 156.5 (s), 162.9 (s), 168.8 (s). Anal. Calculated for C_14_H_10_N_4_OS (MW: 282.32): C, 59.56; H, 3.57; N, 19.85%. Found: C, 59.79; H, 3.81; N, 19.98%.

7. -Methyl-2-(1-methyl-1H-indol-3-yl)-5H-[1,3,4]thiadiazolo[3,2-a]pyrimidin-5-one (**8l**)

Yield: 52%; brown solid; mp: 260 °C; IR (cm^−1^): 1674 (CO). ^1^H NMR (200 MHz, DMSO) δ 3.10 (3H, d, J = 2.6 Hz, CH3), 3.87 (3H, s, CH_3_), 6.28 (1H, d, J = 2.5 Hz, Ar), 7.34 (2H, m, Ar), 7.62 (1H, dd, J = 2.4, 4.1 Hz, Ar), 8.19 (1H, dd, J = 2, 3.9 Hz, Ar), 8.36 (1H, s, Ar). ^13^C NMR (50 MHz, DMSO) δ: 23.7 (q), 23.7 (q), 104.7 (s), 107.2 (d), 111.6 (d), 121.2 (d), 122.6 (d), 123.9 (d), 124.5 (s), 135.2 (d), 137.8 (s), 153.2 (s), 156.4 (s), 162.9 (s), 168.8 (s). Anal. Calculated for C_15_H_12_N_4_OS (MW: MW 296.35): C, 60.79; H, 4.08; N, 18.91%. Found: C, 60.93; H, 4.35; N, 19.22%.

2. -(1H-Indol-3-yl)-7-thiophen-3-yl-[1,3,4]thiadiazolo[3,2-a]pyrimidin-5-one (**8m**)

Yield: 60%; beige solid; mp: 337 °C; IR (cm^−1^): 3301(NH), 1668 (CO). ^1^H NMR (200 MHz, DMSO) δ 6.93 (1H, s, Ar), 7.31 (2H, m, Ar),7.55 (1H, d, J = 3.6 Hz, Ar), 7.69 (1H, d, J = 1.4 Hz, Ar), 7.76 (1H, d, J = 0.8 Hz, Ar), 8.23 (1H, m, Ar) 8.35 (1H, s, Ar), 8.38 (1H, s, Ar), 12.23 (1H, s, NH). ^13^C NMR (50 MHz, DMSO) δ: 103.46 (d), 105.92 (s), 113.11 (d), 121.21 (d), 122.32 (d), 123.94 (d), 124.26 (d), 126.81 (s), 128.16 (d), 130.05 (d), 131.93 (d), 137.29 (s), 139.38 (s), 153.76 (s), 155.27 (s), 157.15 (s), 161.61 (s). Anal. Calculated C_17_H_10_N_4_OS_2_ (MW.: 350.42): C, 58.27; H, 2.88; N, 15.99%. Found: C, 58.53; H, 3.05; N, 16.31%.

2. -(5-Methoxy-1H-indol-3-yl)-7-thiophen-3-yl-[1,3,4]thiadiazolo[3,2-a]pyrimidin-5-one (**8n**)

Yield: 53%; brown solid; mp: 340 °C; IR (cm^−1^): 3101 (NH); 1670 (CO). ^1^H NMR (200 MHz, DMSO) δ 3.86 (3H, s, CH_3_), 6.94–7.00 (2H, m, Ar), 7.48 (1H, d, J = 6.4 Hz, Ar), 7.69–7.80 (3H, m, Ar), 8.34 (2H, d, J = 6.0 Hz, Ar), 12.14 (1H, s, NH). ^13^C NMR (50 MHz, DMSO) δ 56.0 (q), 103.4 (d), 103.5 (d), 105.6 (s), 113.4 (d), 113.8 (d), 125.0 (s), 126.8 (d), 128.0 (d), 128.1 (d), 128.7 (s), 132.0 (d), 132.2 (s), 139.4 (s), 155.2 (s), 155.9 (s), 157.0 (s), 167.4 (s). Anal. Calculated for C_18_H_12_N_4_O_2_S_2_ (MW: 380.44): C, 56.83; H, 3.18; N, 14.73%. Found: C, 56.83; H, 3.18; N, 14.73%.

2. -(5-Bromo-1H-indol-3-yl)-7-thiophen-3-yl-[1,3,4]thiadiazolo[3,2-a]pyrimidin-5-one (**8o**)

Yield: 54%; grey solid; mp: 326 °C; IR (cm^−1^): 3437 (NH); 1674 (CO). ^1^H NMR (200 MHz, DMSO) δ 6.97 (1H, s, Ar), 7.45 (1H, d, J = 8.9 Hz, Ar), 7.55 (1H, d, J = 8.6 Hz, Ar), 7.70 (1H, d, J = 2.3 Hz, Ar), 7.78 (1H, d, J = 3.2 Hz, Ar), 8.27 (1H, s, Ar), 8.37 (1H, s, Ar), 8.48 (1H, s, Ar), 11.93 (1H, s, NH). ^13^C NMR (50 MHz, DMSO) δ: 103.4 (d), 105.5 (s), 114.9 (s), 115.2 (d), 123.3 (d), 125.8 (s), 126.5 (d), 126.8 (d), 128.1 (d), 128.2 (d), 133.2 (d), 136.1 (s), 139.3 (s), 153.4 (s), 155.3 (s), 157.1 (s), 161.5 (s). Anal. Calculated for C_17_H_9_BrN_4_OS_2_ (MW: 429.31): C, 47.56; H, 2.11; N, 13.05%. Found: C, 47.82; H, 2.30; N, 13.28%.

2. -(5-Chloro-1H-indol-3-yl)-7-thiophen-3-yl-[1,3,4]thiadiazolo[3,2-a]pyrimidin-5-one (**8p**)

Yield: 58%; light brown solid; mp: >350 °C; IR (cm^−1^): 3101 (NH); 1668 (CO). ^1^H NMR (200 MHz, DMSO) δ: 7.07 (1H, s, Ar), 7.36 (1H, s, Ar), 7.44–7.79 (3H, m, Ar), 8.33–8.58 (3H, m, Ar), 12.52 (1H, s, NH).^13^C NMR (50 MHz, DMSO) δ: 103.5 (d), 114.8 (d), 115.9 (s), 120.3 (d), 120.9 (s), 124.0 (d), 125.3 (s), 126.8 (d), 127.0 (s), 128.2 (d), 128.2 (d), 129.0 (s), 133.4 (d), 135.9 (s), 139.4 (s), 155.4 (s), 157.1 (s). Anal. Calculated for C_17_H_9_ClN_4_OS_2_ (MW: 384.86): C, 53.05; H, 2.36; N, 14.56%. Found: C, 53.28; H, 2.52; N, 14.80%.

2. -(5-Fluoro-1H-indol-3-yl)-7-thiophen-3-yl-[1,3,4]thiadiazolo[3,2-a]pyrimidin-5-one (**8q**)

Yield: 62%; beige solid; mp: >350 °C; IR (cm^−1^): 3226 (NH), 1670 (CO), ^1^H NMR (200 MHz, DMSO) δ 6.95 (1H, s, Ar), 7.18 (1H, t, J = 8.0 Hz, Ar), 7.57–7.92 (4H, m, Ar), 8.41 (2H, d, J = 3.4 Hz, Ar), 12.38 (1H, s, NH). ^13^C NMR (50 MHz, DMSO) δ: 103.4 (d), 106.0 (d), 106.2 (d), 112.3 (d), 114.5 (s), 124.7 (s), 126.7 (s), 128.1 (2xd), 133.5 (d), 133.9 (d), 139.3 (s), 153.5 (s), 155.3 (s), 157.1 (s), 160.0 (s), 161.5 (s). Anal. Calculated for C_17_H_9_FN_4_OS_2_ (MW: 368.41): C, 55.42; H, 2.46; N, 15.21%. Found: C, 55.68; H, 2.71; N, 15.43%.

2. -(1H-Indol-3-yl)-7-thiophen-3-yl-[1,3,4]thiadiazolo[3,2-a]pyrimidin-5-one (**8r**)

Yield: 63%; grey solid; mp: 340 °C; IR (cm^−1^): 1678 (CO). ^1^H NMR (200 MHz, DMSO) δ 3.90 (3H, s, CH_3_), 6.93 (1H, s, Ar), 7.42–7.29 (3H, m, Ar), 7.62 (1H, d, J = 7.1 Hz, Ar), 7.69 (1H, s, Ar), 7.76 (1H, d, J = 3.9 Hz, Ar), 8.21 (1H, d, J = 5.9 Hz, Ar), 8.38 (1H, s, Ar). ^13^C NMR (50 MHz, DMSO) δ: 33.7 (q), 103.4 (d), 104.7 (s), 110.8 (d), 111.5 (d), 121.2 (d), 122.6 (d), 123.9 (s), 124.5 (s), 126.7 (d), 128.1 (d), 135.2 (d), 137.8 (d), 139.3 (s), 153.2 (s), 155.2 (s), 157.1 (s), 161.4 (s). Anal. Calculated for C_18_H_12_N_4_OS_2_ (MW: 364.44): C, 59.32; H, 3.32; N, 15.37%. Found: C, 59.58; H, 3.60; N, 15.61%.

2. -(5-Methoxy-1-methyl-1H-indol-3-yl)-7-thiophen-3-yl-[1,3,4]thiadiazolo[3,2-a]pyrimidin-5-one (**8s**)

Yield: 56%; brown solid; mp: 323 °C; IR (cm^−1^): 1676 (CO) ^1^H NMR (200 MHz, DMSO) δ 3.85(3H, s, CH_3_), 3.87 (3H, s, CH3), 6.90 (1H, s, Ar), 7,53 (1H, dd, J = 0.9, 1.7 Hz, Ar), 7.69 (2H, m, Ar), 7.76 (1H, s, Ar), 8.30 (1H, s, Ar) 8.30 (1H, s, Ar), 8.32 (1H, d, J = 2.2 Hz, Ar). ^13^C NMR (50 MHz, DMSO) δ: 33.8 (q), 56.0 (q), 103.4 (d), 103.7 (d), 104.3 (s), 112.4 (d), 113.2 (d), 116.2 (d), 125.3 (s), 126.8 (d), 128.1 (d), 135.3 (d), 139.3 (s), 153.2 (s), 153.4 (s), 154.9 (s), 155.1 (s),156.2 (s), 157.0 (s). Anal. Calculated for C_19_H_14_N_4_O_2_S_2_ (MW: 394.47): C, 57.85; H, 3.58; N, 14.20%. Found: C, 57.98; H, 3.80; N, 14.49%.

2. -(5-Bromo-1-methyl-1H-indol-3-yl)-7-thiophen-3-yl-[1,3,4]thiadiazolo[3,2-a]pyrimidin-5-one (**8t**)

Yield: 58%; brown solid; mp: 320 °C; IR (cm^−1^): 1647 (CO). ^1^H NMR (200 MHz, DMSO) δ 3.92 (3H, s, CH_3_), 6.97 (1H, s, Ar), 7.50–7.78 (4H, m, Ar), 7.71 (1H, s, Ar), 8.36 (1H, s, Ar), 8.47 (1H, s, Ar).^13^C NMR (50 MHz, DMSO) δ: 33.99 (q), 103.51 (d), 104.38 (s), 113.89 (d), 115.39 (s), 123.42 (d), 126.03 (s), 126.57 (d), 126.81 (d), 128.18 (d), 128.23 (d), 136.57 (d), 136.72 (s), 139.32 (s), 148.86 (s), 155.31 (s), 157.12 (s), 165.18 (s). Anal. Calculated for C_18_H_11_BrN_4_OS_2_ (MW: 443.34): C, 48.76; H, 2.50; N, 12.64%. Found: C, 48.92; H, 2.69; N, 12.80%.

2. -(5-Chloro-1-methyl-1H-indol-3-yl)-7-thiophen-3-yl-[1,3,4]thiadiazolo[3,2-a]pyrimidin-5-one (**8u**)

Yield: 52%; brown solid; mp > 350 °C; IR (cm^−1^): 1668 (CO). ^1^H NMR (200 MHz, DMSO) δ: 3.90 (3H, s, CH_3_), 6.93 (1H, s, Ar), 7.39 (1H, d, J = 8.2 Hz, Ar), 7.51–7.75 (3H, m, Ar), 7.75 (1H, dd, J = 1.0, 1.1 Hz, Ar), 8.18 (1H, s, Ar), 8.33 (1H, s, Ar), 8.43 (1H, d, J = 4.6 Hz, Ar). ^13^C NMR (50 MHz, DMSO) δ 33.9 (q), 103.4 (d), 112.1 (s), 113.4 (d), 115.3 (s), 120.3 (d), 123.4 (d), 123.9 (d), 125.4 (s), 125.7 (s), 126.7 (d), 127.3 (s), 128.19 (d), 136.4 (s), 136.6 (d), 139.3 (s), 155.3 (s), 157.0 (s). Anal. Calculated for C_18_H_11_ClN_4_OS_2_ (MW: 398.89): C, 54.20; H, 2.78; N, 14.05%. Found: C, 54.41; H, 2.95; N, 14.38%.

2. -(5-Fluoro-1-methyl-1H-indol-3-yl)-7-thiophen-3-yl-[1,3,4]thiadiazolo[3,2-a]pyrimidin-5-one (**8v**)

Yield: 61%; grey solid; mp >350 °C; IR (cm^−1^): 1656 (CO). ^1^H NMR (200 MHz, DMSO) δ 3.92 (3H, s, CH_3_), 6.92 (1H, s, Ar), 7.24 (1H, s, Ar), 7.68–7.91 (4H, m, Ar), 8.40 (2H, d, J = 2.2 Hz Ar). ^13^C NMR (50 MHz, DMSO) δ 34.0 (q), 103.4 (d), 104.7 (s), 105.5 (s), 106.0 (d), 111.9 (d), 112.3 (d), 113.1 (d), 124.9 (s) 126.7 (d), 128.1 (d), 134.5 (s), 136.7 (d), 139.3 (s), 153.0 (s), 155.2 (s), 157.0 (s), 160.7 (s). Anal. Calculated for C_18_H_11_FN_4_OS_2_ (MW: 382.43): C, 56.53; H, 2.90; N, 14.65%. Found: C, 56.80; H, 2.99; N, 14.88%.

### 3.2. Biology

#### 3.2.1. Determination of Minimum Inhibitory Concentrations (MICs)

The MICs were determined via the microdilution method. The serial dilutions of each compound were made in Mueller–Hinton broth (MH) in a 96-well plate, starting from a stock solution of 5 mg/mL in Dimethyl Sulfoxide (DMSO). A series of concentrations of each extract in MH medium, ranging between 100 and 0.7 µg/mL, were tested. The compounds were tested against reference strains *S. aureus* ATCC 25923, *P. aeruginosa* ATCC 15442, E. faecalis ATCC 29212, and *E. coli* ATCC 25922. A bacterial suspension in 0.9% NaCl was prepared until 10^6^ colony-forming units (CFU)/mL were obtained by two-fold dilution of the bacteria, grown at 37 °C for 24 h on Tryptic Soy Agar (TSA) [24]. To each well of a 96-well plate containing the compound, 10 µL of a bacterial suspension was added. A positive control (bacterial strains in the medium without compounds) and a negative control (medium without inoculum) were also included in the 96-well plate. Moreover, a substance control was added to evaluate the absorbance of compounds, consisting only of the compound solution, without bacterial inoculum. The 96-well plates were incubated at 37 °C for 24 h and MICs were read by a microplate spectrophotometer (GloMax^®^-Multi Detection System, Promega) as the lowest concentration of the extract whose o.d., read at 570 nm, was comparable with the negative control wells (broth without bacterial inoculum) [25].

#### 3.2.2. Antibiofilm Activity

The above-mentioned bacterial strains were incubated in 5 mL of Tryptic Soy Broth (TSB) containing 2% (*w*/*v*) glucose at 37 °C for 24 h. After the incubation time, 2.5 μL of each microbial suspension was placed into each well of flat-bottom 96-well loaded with 200 μL of TSB with glucose 2% [26]. Aliquots at sub-MIC concentrations of each compound, ranging from 80 to 10 μg/mL, were directly added to the wells. Plates were incubated at 37 °C for 24 h. After biofilm growth, wells were washed twice with sterile NaCl 0.9% and sessile biomass stained with 100 μL of 0.1% crystal violet solution for 30 min at 37 °C [27]. Then, surplus solution was discharged, and the plate was washed twice using tap water. A quantity of ethanol (200 μL) was added to each stained well to solubilize the dye bound to biofilm for 10 min. OD was read at a wavelength of 540 nm using a plate reader (Glomax Multidetection System TM297 Promega, Milano, Italy). The experiments were performed at least in triplicate, and three independent experiments were performed. The percentage of inhibition was calculated using the following formula:

% of inhibition = (OD growth control − OD sample)/OD growth control) × 100

BIC_50_ (the concentration at which the percentage of inhibition of biofilm formation is equal to 50%) [28]. All data points are expressed as means ± SDs of three separate experiments performed in triplicate. A statistical analysis of the treated samples was preformed using *t*-test, *p* < 0.005.

#### 3.2.3. Effect of Compounds against Preformed Biofilm Biomasses

The reference strains *S. aureus* ATCC 25923, *P. aeruginosa* ATCC 15442, *E. faecalis* ATCC 29212, *E. coli* ATCC 25922, *S. epidermidis* ATCC 12228, *S. hominis* ATCC 27844, *P. acnes* ATCC11827, *S. agalactiae* ATCC 10231, and *C. albicans* ATCC 10231 were used in the preformed biofilm tests. The bacterial strains were cultured as described; fungal *C. albicans* strain was cultured aerobically on Sabouraud broth with glucose 2% or agar medium. Biofilms were allowed to form in each well of a 96-well microtiter plate, as described above. After 24 h, the planktonic cells were gently removed by aspiration, and the plate was washed with 200 μL of NaCl 0.9%. Compounds were added on each well at concentrations ranging from 200 μg/mL to 10 μg/mL, and the plate was incubated for 24 h at 37 °C. After incubation time, crystal violet-staining was performed to assess the biofilm biomass, as described in Section 3.2.2. All data points are expressed as means ± SDs of three separate experiments performed in triplicate. Statistical analysis among treated samples was preformed using *t*-test, *p* < 0.005.

#### 3.2.4. In Vivo Anti-Infective Activity and Toxicity Evaluation of Compound **8j**

##### Insect Rearing and Preparation

Larvae of greater wax moth *G*. *mellonella* L. (Lepidoptera: Pyralidae) were reared on a natural diet–honeybee nest debris at 30 °C in the dark. The last instar creamy color larvae, with an average weight of 399 mg (±110 mg) and approximately 2.5 cm long, were selected for this study. Before setting the experiments, larvae health and quality were assessed.

Before in vivo bioassay, the larvae were washed with distilled water and then immersed briefly in 70% (*v*/*v*) ethanol to sterilize their surfaces.

##### In Vivo Bioassay

To evaluate the property of compound **8j** to protect larvae of *G*. *mellonella* from infection caused by pathogenic bacteria, an in vivo experiment was performed.

*S. aureus* ATCC 29213 and *P. aeruginosa* ATCC 15442 (10 μL of live bacteria corresponding to 2.8 × 10^4^ cfu/mL) were injected into the group of larvae. Simultaneously, the larvae were inoculated with single treatment dose of 1 mg/kg of the compound dissolved in NaCl 0.9%. Two uninfected group controls were set up, one untreated and the other injected with NaCl 0.9% (*v*/*v*) containing the same solvents used to dissolve the tested compound. Two infected positive group controls were set up, inoculating *S. aureus* or *P. aeruginosa* (2.8 × 10^4^ cfu/mL) without an antibacterial agent. One group of larvae was inoculated with a single treatment dose of 1 mg/kg of the compound **8j**. Three replicates of four larvae for each group were disposed; a total of 84 larvae were used, including a group without any type of treatment.

Larvae were inoculated by injection between the left third and fourth prolegs using a 100 μL Hamilton syringe: the first puncture was performed on the left side and the second one on the right side to avoid stress on the same side. Following the inoculation, larvae were incubated at 37 °C in the dark and monitored after 6, 24, and 48 h for mortality and melanization (dark pigmentation) over 96 h unless otherwise stated.

##### Insect Data Analysis

All statistical analyses were performed using Statistica 6.0 (StatSoft, Tulsa, OK, USA) with a significance level of α = 0.05. The corrected mortalities of larvae were calculated using Abbott’s formula. Significant differences between values were determined by one-way ANOVA, and differences between different treatment groups and the reference control groups were determined via statistical analysis and Tukey’s post-test. Simultaneously, the Kaplan–Meier survival analysis with Mantel—Cox log-rank test was used to construct the survival curves for *G*. *mellonella*.

## 4. Conclusions

In summary, the structural manipulation of a library of nortopsentin analogues with antibiofilm properties allowed for a new series of 22 [1,3,4]thiadiazolo[3,2-a]pyrimidin-5 to be obtained as potent dispersal agents. These compounds demonstrate potential in the development of potent anti-virulence agents capable of disrupting biofilm architecture in a wide range of pathogens, including Gram-positive and Gram-negative bacteria. Importantly, derivatives **8a**–**v** maintain the anti-virulence profile of the thiazole nortopsentin analogues, primarily targeting the biofilm mode of life without exerting substantial effects on microbial viability.

The replacement of the thiazole central core of derivatives **1** with the [1,3,4]thiadiazolo[3,2-a]pyrimidin-5-one scaffold resulted in a notable shift in the mechanism of biofilm inhibition, transitioning from anti-adhesion activity to dispersal activity. Furthermore, these newly synthesized compounds exhibited an expanded spectrum of action, displaying increased potency against Gram-negative pathogens compared to the previous series. Moreover, the results revealed that the presence of a phenyl group at position 7 of the thiadiazopyrimidinone nucleus is advantageous compared to methyl and thiophene rings in terms of anti-biofilm activity. Of particular interest, compound **8j** has demonstrated, both in vitro and in vivo, remarkable activity and tolerability. It holds promise as an attractive lead compound for the development of a novel class of potent dispersal agents with broad-spectrum activity. Compound **8j** holds potential for effectively treating serious chronic infections mediated by biofilms.

## Figures and Tables

**Table 1:** New [1,3,4]thiadiazolo[3,2-a]pyrimidin-5-one derivatives **8a**–**v**.

Compound	R	R^1^	R^2^
**8a**	H	H	Ph
**8b**	OCH_3_	H	Ph
**8c**	Br	H	Ph
**8d**	Cl	H	Ph
**8e**	F	H	Ph
**8f**	H	CH_3_	Ph
**8g**	OCH_3_	CH_3_	Ph
**8h**	Br	CH_3_	Ph
**8i**	Cl	CH_3_	Ph
**8j**	F	CH_3_	Ph
**8k**	H	H	CH_3_
**8l**	H	CH_3_	CH_3_
**8m**	H	H	3-thiophenyl
**8n**	OCH_3_	H	3-thiophenyl
**8o**	Br	H	3-thiophenyl
**8p**	Cl	H	3-thiophenyl
**8q**	F	H	3-thiophenyl
**8r**	H	CH_3_	3-thiophenyl
**8s**	OCH_3_	CH_3_	3-thiophenyl
**8t**	Br	CH_3_	3-thiophenyl
**8u**	Cl	CH_3_	3-thiophenyl
**8v**	F	CH_3_	3-thiophenyl

**Table 2:** Antimicrobial activity of compounds tested against reference bacterial free-living strains.

Compound	MIC (µg/mL)			
	Pathogen			
	*S*. *aureus* ATCC 25923	*P. aeruginosa* ATCC 15442	*E*. *coli* ATCC 25922	*E*. *faecalis* ATCC 29212
**8a**	>100	>100	>100	>100
**8b**	>100	>100	>100	>100
**8c**	>100	>100	>100	>100
**8d**	>100	>100	>100	>100
**8e**	50	>100	>100	>100
**8f**	100	>100	>100	>100
**8g**	>100	>100	>100	>100
**8h**	>100	>100	>100	>100
**8i**	>100	>100	>100	>100
**8j**	>100	>100	>100	50
**8k**	50	>100	>100	25
**8l**	50	>100	>100	>100
**8m**	>100	>100	>100	>100
**8n**	>100	>100	>100	>100
**8o**	>100	>100	>100	>100
**8p**	>100	>100	>100	>100
**8q**	>100	>100	>100	>100
**8r**	>100	>100	>100	>100
**8s**	>100	>100	>100	>100
**8t**	>100	>100	>100	>100
**8u**	>100	>100	>100	>100
**8v**	>100	>100	>100	>100

**Table 3:** Inhibition of biofilm formation, expressed as IBF (% at 100 µg/mL) and BIC_50_, µg/mL (µM).

Compounds	IBF (%, 100 µg/mL)	BIC_50_ µg/mL (µM)	Pathogen
**8c**	51 (±5.0)	10.4 (24.5)	*P*. *aeruginosa* ATCC 15442
**8e**	54 (±5.2)	18.2 (50.2)	*S*. *aureus* ATCC 25923
**8f**	75 (±7.3)	7.6 (21.3)	*S*. *aureus* ATCC 25923
**8j**	60 (±5.9)	14.4 (38.4)	*P. aeruginosa* ATCC 15442
	70 (±6.8)	6.3 (16.7)	*S*. *aureus* ATCC 25923
	64 (±6.2)	6.1 (16.2)	*E*. *coli* ATCC 25922
**8k**	64 (±6.1)	21.1 (75.8)	*S*. *aureus* ATCC 25923
	57 (±5.5)	6.4 (22.6)	*E. faecalis* ATCC 29212
**8l**	53 (±5.1)	14.5 (48.9)	*P. aeruginosa* ATCC 15442

**Table 4:** Mean values and standard deviation (SD) of anti-biofilm activity of compounds **8a**–**l** against 24 h preformed biofilms of *S. aureus* ATCC 25923, *P. aeruginosa* ATCC 15442, and *E. coli* ATCC 25922, expressed as inhibition percentage (%) at the screening concentration of 200 µg/mL.

Compounds	*S*. *aureus* ATCC 25923	*P*. *aeruginosa* ATCC 15442	*E*. *coli* ATCC 25922
**8a**	69 (±6.9)	65 (±6.5)	53 (±5.3)
**8b**	71 (±7.1)	59 (±5.9)	75 (±7.5)
**8c**	58 (±5.8)	67 (±6.7)	73 (±7.3)
**8d**	66 (±6.0)	63 (±6.3)	63 (±6.3)
**8e**	51 (±5.0)	62 (±6.1)	65 (±6.5)
**8f**	69 (±6.9)	54 (±5.4)	67 (±6.7)
**8g**	72 (±7.0)	68 (±6.7)	73 (±7.3)
**8h**	66 (±6.1)	77 (±7.3)	70 (±7.0)
**8i**	69 (±6.9)	67 (±6.7)	59 (±5.7)
**8j**	60 (±5.9)	69 (±6.9)	68 (±6.8)
**8k**	66 (±6.6)	73 (±7.3)	70 (±7.0)
**8l**	65 (±6.5)	71 (±7.0)	76 (±7.3)

## Data Availability

The data presented in this study are available on request from the corresponding author.
